# A Short Lecture Delivered to the Students of the Atlanta Medical College, on Influenza

**Published:** 1897-02

**Authors:** W. S. Kendrick

**Affiliations:** Atlanta, Ga.; Professor of Principles and Practice of Medicine and Clinical Medicine


### A SHORT LECTURE DELIVERED TO THE STUDENTS OF THE ATLANTA MEDICAL COLLEGE ON INFLUENZA.

By W. S. KENDRICK, M. D., ATLANTA, GA.

Professor of Principles and Practice of Medicine and Clinical Medicine.

*Gentlemen*: The next of the special diseases that will engage our attention this morning is one prevailing very largely at this time, not only in this city, but throughout the length and breadth of the land. Influenza, or la grippe, as it is most usually termed, is one of the specific, acute, infectious diseases, characterized by a train of symptoms, and by constitutional disturbances, different from almost any other disease in the same group. It is a disease almost forgotten until the winter of 1888, when it began in St. Petersburg, spread rapidly over Europe, reached the United States in a few weeks, and by

January, 1889, was epidemic almost throughout this entire country. Since that time a yearly visitation during the winter has occurred, sometimes severe, and at other times lighter. Probably none have been so pronounced as the first, which was exceedingly severe and many succumbed to its fearful ravages. It follows along great highways of travel, appearing in the largest cities usually first, and then finding, from those centers of population, its way into more sparsely settled districts. It attacks persons in different ways, the most frequent of which is a catarrhal condition of the respiratory organs. Some times the mucous membrane lining the naso-pharynx alone is involved. At other times the disease attacks the lining membrane of trachea and larger tubes, and again it finds its way down into the smaller tubes, which latter condition is much more serious than either of the others. In other cases the respiratory organs escape almost entirely, and the disease spends its force on the gastro-intestinal tract, producing great functional disturbance of the same. Another form of the disease, which is exceedingly painful and more depressing, is an affection of the nervous and muscular systems, with the catarrhal condition of the respiratory organs and the disturbance of the gastro-intestinal tract largely absent. It is a typical disease, and during an epidemic there is no trouble whatever in making an accurate diagnosis. It is due to a specific poison, the bacillus of Pfeiffer, which is present in great numbers, particularly in the discharges from the nose and throat. It is not an ordinary cold. It has no specially direct relations with an ordinary cold, but it is due to altogether different causes, to its own specific cause. In the ordinary cases there are scarcely any pathological changes prominent outside of the complications and sequelæ.

The symptoms of the disease vary very greatly. In one case they are very slight, in another exceedingly severe. In the first instance the patient emerges from the condition in a very few days, in the other the most pronounced constitutional symptons are present, causing the greatest anxiety and distress. In the majority of cases the disease is ushered in by a chill, or by chilly sensations recurring frequently, followed by a fever of varying intensity, sometimes exceedingly slight, and at other times ranging from 101 to 104, or in extreme cases, even to 105 degrees. The pulse is usually more or less rapid, and in many cases where complications exist, or where the nervous system is profoundly impressed, the heart's action may become very rapid. Outside of the chills and fever, the most pronounced constitutional symptoms are pains about the head, face, and eyes about the muscles of the neck, back and loins, and with great muscular soreness over the entire body. In some cases again, the joints are exceedingly painful and tender, a general hyperæsthesia exists, and the patients feel as if they were rapidly going into an attack of acute rheumatism. The symptoms accompanying the various varieties are well marked. The coryza, the pharyngitis, the tracheitis, and bronchitis are all conditions which are more or less distressing, and which can not be mistaken. Whatever variety of the disease is present, there are always great depression of spirits and a debility and exhaustion out of all proportion to the catarrhal condition of the respiratory organs or any other conditions that may be present. I know of no acute condition which produces such marked depression, and accompanied by such slow recovery, many cases feeling that they will not recover at all.

The duration of the disease in the majority of cases lasts only a few days. In others, without complications, the catarrhal condition of the respiratory organs may continue for a longer period of time. Relapses are of frequent occurrence, and in many instances they are much more severe than the original attack. They are particularly liable to occur if the patient unduly exposes himself before recovery has fully taken place. I have seen a number of such instances during the present prevailing epidemic.

The complications are various, but are usually associated with the respiratory organs. Pneumonia is probably the most frequent and fatal of all the complications. Pleuritis peri- and endocarditis also occur. It is often the case that patients in private practice, and a large number of cases applying for treatment at our general medical clinics in this building, date their ailments and failing health to some previous attack of influenza. It is frequently the case that those suffering from general debility and predisposed to certain chronic diseases, notably tuberculosis, succumb in the course of time to those special conditions, influenced largely by a previous attack of the disease under consideration. For these reasons, each epidemic leaves in its widespread track many victims that would not have been attacked by intercurrent affections had not their vitality, their resistive forces, been lowered by a previous attack of influenza.

A latent endocarditis, a chronic nephritis, a tuberculosis and various other conditions, emerge, as it were, from their hiding-places, after an epidemic, and crowd their unwilling victims into the grave.

During an attack, if the temperature following a rigor should rise to 104° or 105°, an dremain at about that point for two or three days, or longer, we should always be exceedingly guarded in our prognosis, for some complication is almost invariably present. Pneumonia, under those conditions, is the one most to be considered, as it is the most frequent. A recent case under my care, a typical illustration, showed the following symptoms, viz.: Disease ushered in by chill, followed by moderately low fever, slow pulse, but with most violent catarrhal symptoms about the nose and throat. That condition lasted eight or ten days, when the symptoms disappeared and the patient was dismissed. During convalescence she was seized with a chill, followed by a temperature of 105°, with the most pronounced symptoms of depression. The pain in the head was severe, the pulse 136, and maintained its height continuously. Temperature ranged from 103° to 104° for a week. I was positive, from the constitutional symptoms, that pneumonia was present, but was unable to detect the physical signs of the same. I examined carefully, time and again, without being able to find anything specially abnormal about the respiration. On the fifth day I found moist rales over the lower anterior surface of the right lung, and after the most scrutinizing and careful examination, tubular breathing was heard on the upper posterior surface of the same side. It required both the ear and stethoscope on the skin to find the abnormal sounds. Percussion gave resonance. Here was a typical case of solidification of lung tissue, but not typical of lobar pneumonia. It was a case of deep central pneumonia surrounded by lung tissue, which was not solidified, hence the resonance on percussion and delay in finding bronchial breathing. The main reason that pneumonia is so exceedingly fatal in la grippe is due to the fact that the original disease is a distinctive condition from the pneumonia, and depresses the general system to a great degree. When followed by pneumonia, which of itself is a very grave disease, the two combined depress and poison the system to such an extent that resistance is lost, the heart's action becomes weakened, vital forces give way and a large percentage of that class of patients die.

The prognosis depends largely on the age, the physical condition of the patient, and the complications. The debilitated, the old and the feeble are stricken down in many instances, while the young and those whose resistive forces are in good condition often recover. In the latter class of subjects, rid of the complications, nearly all get well.

The treatment varies according to the condition of the patient. A case with the pronounced catarrhal conditions of the respiratory organs must have appropriate treatment for same. If the gastro-intestinal variety is present, the same idea will hold good. If a bronchitis, we must apply such treatment as is used for that affection. Opium in moderate quantities, preparations of ipecac, expectorants if needed, and various other remedies which may be required for the comfort of the patient. Ipecac in some form is probably the best remedy for acute conditions of the bronchial tubes outside of small doses of opium. Absolute cleanliness as far as can be done, should be practiced, as infection comes largely from the secretions of the nose and throat. In the gastro-intestinal variety accompanied with nausea and vomiting, small doses of calomel combined with rhubarb or salines, will be frequently beneficial at the beginning. Whatever treatment is adopted, it is probably best to precede it with some mercurial or saline. For the intense headache, great muscular pains and discomfort, preparations of opium are among the very best. My plan in those cases, if the pain is exceedingly severe, is to give a hypodermic injection of from one-eighth to one-quarter of a grain of sulphate of morphia and atropia, at once, or, if the patient is not suffering too much, leave off the morphine and give about the following prescription.

To each capsule, from one to two grains bisulphate of quinine; two to three grains of phenacetine; $\frac{1}{8}$ grain sulphate of codei, or two grains of pulverized Dovers; and from one-half to a grain of powdered camphor.

One of these capsules taken every two hours until the pain becomes less, and then three or four times daily, will give the patient great comfort and ease. Any other preparation of opium or antipyretic might be just as efficaciously used. The general treatment should be supportive. Alcholic stimulants in a moderate degree, followed by tonics later on, and an exceedingly nutritious diet, will usually tide our patients over the severest attacks, unless grave complications appear. If complication, they must receive such treatment as will be appropriate to each individual case and condition, and of which I have attempted to explain so often.

---

### HOMEOPATHIC (?) TREATMENT OF NIGHT SWEATS.

Camphoric acid, in doses of 5 to 10 grains, is the most generally useful remedy I have ever used. I only give one dose in the evening, rarely two. The sweats often cease at once under its use.

Hydrastis in doses of 5 to 20 drops of the tincture always succeeds when the sweats are the result of debility from exhausting diseases, as typhoid fever, la grippe, or extreme exhaustion from overwork. The dose is given several times during the day.

In some chronic cardiac diseases with very low arterial tension, no drug gives better results than digitalis in doses of 5 drops four times a day.

Cimicifuga in small doses often arrests the sweats of rheumatism, while our old drug china is almost specific when profuse sweats occur on going to sleep.

I cured one obstinate case of night sweats after influenza with pyrotoxin, 1-100 grain three times a day.—E. M. Hale, in the *Hahnemannian Monthly*.

